# Assessment of Magnetic Resonance Imaging Changes and Functional Outcomes Among Adults With Severe Herpes Simplex Encephalitis

**DOI:** 10.1001/jamanetworkopen.2021.14328

**Published:** 2021-07-27

**Authors:** Benjamine Sarton, Pierre Jaquet, Djida Belkacemi, Etienne de Montmollin, Fabrice Bonneville, Charline Sazio, Aurelien Frérou, Marie Conrad, Delphine Daubin, Russell Chabanne, Laurent Argaud, Frédéric Dailler, Noëlle Brulé, Nicolas Lerolle, Quentin Maestraggi, Julien Marechal, Pierre Bailly, Keyvan Razazi, Francois Mateos, Bertrand Guidet, Albrice Levrat, Vincent Susset, Alexandre Lautrette, Jean-Paul Mira, Ahmed El Kalioubie, Alexandre Robert, Alexandre Massri, Jean François Albucher, Jean Marc Olivot, Jean Marie Conil, Lila Boudma, Jean-François Timsit, Romain Sonneville, Stein Silva

**Affiliations:** 1 Critical Care Unit, University Hospital of Purpan, Toulouse, France; 2 Toulouse NeuroImaging Center, Unité Mixte de Recherche 1214, Institut National de la Santé et de la Recherche Médicale, Université Paul Sabatier, Toulouse, France; 3 Department of Intensive Care Medicine and Infectious Diseases, Bichat-Claude Bernard University Hospital, Paris, France; 4 Department of Neuroradiology, University Hospital of Purpan, Toulouse, France; 5 Critical Care Unit, University Hospital of Pellegrin, Bordeaux, France; 6 Critical Care Unit, University Hospital of Rennes, Rennes, France; 7 Critical Care Unit, Regional and University Hospital of Nancy, Nancy France; 8 Critical Care Unit, University Hospital of Montpellier, Montpellier, France; 9 Critical Care Unit, University Hospital Gabriel Montpied, Clermont Ferrand, France; 10 Critical Care Unit, University Hospital Edouard Herriot, Hospices Civils of Lyon, Lyon, France; 11 Neurological Critical Care Unit, Hospital Pierre Wertheimer, Hospices Civils of Lyon, Lyon, France; 12 Critical Care Unit, University Hospital of Nantes, Nantes, France; 13 Critical Care Unit, University Hospital of Angers, Angers, France; 14 Critical Care Unit, University Hospital Hautepierre of Strasbourg, Strasbourg, France; 15 Critical Care Unit, University Hospital La Miletrie, Poitiers, France; 16 Critical Care Unit, Regional University Hospital La Cavale Blanche, Brest, France; 17 Critical Care Unit, University Hospital of Henri Mondor, Créteil, France; 18 Critical Care Unit, Regional Hospital of Saint Brieuc, Saint Brieuc, France; 19 Critical Care Unit, University Hospital of Saint Antoine, Paris, France; 20 Critical Care Unit, University Hospital of Annecy Genevois, Epagny Metz-Tessy, France; 21 Critical Care Unit, Regional Hospital of Chambery, Chambery, France; 22 Critical Care Unit, University Hospital of Clermont-Ferrand, Clermont-Ferrand, France; 23 Critical Care Unit, University Hospital Cochin, Paris, France; 24 Critical Care Unit, University Hospital Salengro, Lille, France; 25 Critical Care Unit, University Hospital of Nice, Nice, France; 26 Critical Care Unit, Regional Hospital of Pau, Pau, France; 27 Department of Neurology, University Hospital of Purpan, Toulouse, France; 28 Critical Care Unit, University Hospital of Rangueil, Toulouse, France; 29 Laboratory for Vascular Translational Science, Sorbonne Paris Cité, Unité Mixte de Recherche 1148, Institut National de la Santé et de la Recherche Médicale, Paris Diderot University, Paris, France

## Abstract

**Importance:**

Current guidelines recommend brain magnetic resonance imaging (MRI) for clinical management of patients with severe herpes simplex encephalitis (HSE). However, the prognostic value of brain imaging has not been demonstrated in this setting.

**Objective:**

To investigate the association between early brain MRI data and functional outcomes of patients with HSE at 90 days after intensive care unit (ICU) admission.

**Design, Setting, and Participants:**

This multicenter cohort study was conducted in 34 ICUs in France from 2007 to 2019 and recruited all patients who received a clinical diagnosis of encephalitis and exhibited cerebrospinal fluid positivity for herpes simplex virus DNA in the polymerase chain reaction analysis. Data analysis was performed from January to April 2020.

**Exposures:**

All patients underwent a standard brain MRI during the first 30 days after ICU admission.

**Main Outcomes and Measures:**

MRI acquisitions were analyzed by radiologists blinded to patients’ outcomes, using a predefined score. Multivariable logistic regression and supervised hierarchical classifiers methods were used to identify factors associated with poor outcome at 90 days, defined by a score of 3 to 6 (indicating moderate-to-severe disability or death) on the Modified Rankin Scale.

**Results:**

Overall, 138 patients (median [interquartile range {IQR}] age, 62.6 [54.0-72.0] years; 75 men [54.3%]) with an admission median (IQR) Glasgow Coma Scale score of 9 (6-12) were studied. The median (IQR) delay between ICU admission and MRI was 1 (1-7) days. At 90 days, 95 patients (68.8%) had a poor outcome, including 16 deaths (11.6%). The presence of fluid-attenuated inversion recovery MRI signal abnormalities in more than 3 brain lobes (odds ratio [OR], 25.71; 95% CI, 1.21-554.42), age older than 60 years (OR, 7.62; 95% CI, 2.02-28.91), and the presence of diffusion-weighted MRI signal abnormalities in the left thalamus (OR, 6.90; 95% CI, 1.12-43.00) were independently associated with poor outcome. Machine learning models identified bilateral diffusion abnormalities as an additional factor associated with poor outcome (34 of 39 patients [87.2%] with bilateral abnormalities had poor outcomes) and confirmed the functional burden of left thalamic lesions, particularly in older patients (all 11 patients aged >60 years had left thalamic lesions).

**Conclusions and Relevance:**

These findings suggest that in adult patients with HSE requiring ICU admission, extensive MRI changes in the brain are independently associated with poor functional outcome at 90 days. Thalamic diffusion signal changes were frequently observed and were associated with poor prognosis, mainly in older patients.

## Key Points

**Question:**

Are early brain changes seen on magnetic resonance imaging (MRI) associated with functional outcomes among adult patients with severe herpes simplex encephalitis (HSE)?

**Findings:**

In adult patients with HSE, extensive brain changes seen on MRI during the first month after intensive care unit admission were independently associated with poor functional outcome at 90 days. Thalamic diffusion signal changes were frequently observed and were associated with poor prognosis, mainly in older patients.

**Meanings:**

These findings suggest that, among adult patients with severe HSE, early MRI data were associated with neurological outcomes, both in terms of brain lesion extension and critical focal MRI signal changes.

## Teaser

This cohort study investigates the association between early collected magnetic resonance imaging (MRI) data and functional outcomes 90 days after intensive care unit admission among adults with severe herpes simplex encephalitis.

## Introduction

Herpes simplex encephalitis (HSE) is the most frequently identified cause of sporadic necrotizing encephalitis worldwide.^1,2,3^ In the absence of treatment, the prognosis of HSE is extremely poor, with a mortality rate of approximately 70%.^4^ Improvement in diagnostic techniques and the advent of acyclovir treatment have decreased the mortality rate to 15%, but many patients are still left with substantial disability.^5,6,7,8^ The identification of early and reliable indicators of outcomes in patients with severe HSE might constitute a game-changing factor, leading to more personalized intervention strategies. However, data on outcomes of the most severe cases of HSE are very limited, and previous studies^9,10,11^ about HSE neuroprognostication had limitations, including small samples and single-center designs.

Current guidelines^12^ outline the usefulness of brain magnetic resonance imaging (MRI) in the management of patients with suspected HSE, irrespective of severity. MRI enables fast diagnosis and permits disentangling of HSE from its mimics.^13,14^ Typical radiological MRI findings in HSE are the presence of asymmetrical changes in signal intensities in the mesial temporal lobes, inferior frontal lobes, and insula.^15^ In addition, it has been reported that MRI allows for identification early cerebrovascular complications in severe HSE cases.^16^ Nevertheless, the prognostic value of MRI in this challenging clinical setting is still a moot issue. To our knowledge, only a few studies have systematically investigated neuroimaging in severe HSE. Most of them are case reports that provided contradictory results regarding the prognostic value of MRI in this setting.^9,10^ Most of the published cohort studies were small and did not find a significant association between MRI findings and clinical outcomes.^9,10,11^ To our knowledge, only 1 large multicenter cohort study^17^ has recently suggested that brain lesion extension on initial MRI was associated with worse outcomes but, unfortunately, this information was not available for all patients and was gathered from nonstandardized radiological assessments extracted from patients’ medical records and reviews. Overall, despite its potential clinical usefulness, we clearly lack data on the value of early brain MRI data for the neuroprognostication of patients with HSE.

In this large, multicenter cohort study of patients with severe HSE, we aimed to investigate the association between early MRI data and patients’ functional outcomes at 90 days after intensive care unit (ICU) admission. We make the hypotheses that there is a significant association between the extension of HSE-related brain lesions and patients’ functional outcome, the detection of MRI focal lesions within brain structures known to be critical for higher-order cognitive processes (eg, frontal lobes or thalamus) is associated with a poor neurological outcome, and different MRI sequences provide complementary diagnostic information.

## Methods

### Study Design

This multicenter cohort study was conducted in in 34 ICUs in France, as part of the ENCEPHALITICA Study,^17,18^ between 2007 and 2019. We retrospectively reviewed the electronic medical records of all patients who received a clinical diagnosis of encephalitis (*International Classification of Diseases, Ninth Revision,* code 054.3) and exhibited cerebrospinal fluid (CSF) positivity for herpes simplex virus (HSV) DNA in the polymerase chain reaction analysis. All medical records were reviewed by investigators (B.S. and P.J.). Brain MRI was performed for all patients. The ethical committee of the French Society of Intensive Care Medicine approved the study and waived the requirement for informed consent because the data were deidentified and the study posed minimal risk to participants. This study follows the Strengthening the Reporting of Observational Studies in Epidemiology (STROBE) reporting guideline.

### Patients

Patients were included if they fulfilled the following criteria: ICU admission (and length of stay >24 hours) with possible acute encephalitis and a CSF polymerase chain reaction test positive for HSV DNA during hospitalization. Possible acute encephalitis was defined according to guidance provided by international guidelines^19^ and corresponded to an acute change in mental status or behavior lasting 24 hours or longer, with at least 2 of the following manifestations: fever within 72 hours before or after the presentation, generalized or partial seizures, new onset of focal neurological findings, lumbar puncture with CSF white blood cell (ie, leukocyte) count greater than or equal to 5 cells/μL (to convert to cells ×10^9^/L, multiply by 0.001), and neuroimaging or electroencephalogram abnormalities suggestive of encephalitis. Exclusion criteria were brain MRI not performed or performed more than 30 days after ICU admission, preexisting neurological disease that could interfere with brain MRI analysis (eg, brain tumor or severe traumatic brain injury sequelae), poor-quality MRI (eg, metal and motion artifacts), and missing data on functional outcome at 90 days.

### Collected Clinical and Laboratory Data

The patient’s history, clinical, laboratory, and brain electrophysiologic data were gathered from medical records. Baseline health status before ICU admission was graded by the Knaus score (classes A-D, with A denoting normal health status, B denoting moderate activity limitations, C denoting several activity limitation due to chronic disease, and D denoting being bedridden).^20^ The Simplified Acute Physiology Score II (range, 0-163, with higher scores denoting higher severity of disease and increased risk of mortality) and the Sequential Organ Failure Assessment score (range, 0-24, with higher scores denoting higher degree of organ dysfunction during patient’s ICU stay) were calculated within the first 24 hours after admission.^21,22^ Mental status at ICU admission was graded using the Glasgow Coma Scale (GCS; range, 3-15), with a coma defined as a GCS score less than 8.^23^ Immunodepression was defined as the long-term (>3 months) use of steroids, the use of other immunosuppressant drugs, solid-organ transplantation, solid tumors requiring chemotherapy in the last 5 years, hematological malignant entity (regardless of time since diagnosis and receiving treatment), or AIDS.

### Radiological Examination

MRI acquisitions performed within the first month after ICU admission were considered for examination. MRI acquisitions were performed with 3-T or 1.5-T MRI units using T1-weighted, T2-weighted, gradient-echo T2*-weighted, fluid-attenuated inversion recovery (FLAIR), and diffusion-weighted imaging (DWI) sequences. All Digital Imaging and Communication in Medicine images were transmitted to the workstation and picture archiving and communication systems of Toulouse NeuroImaging Center for multiplanar reconstruction postprocessing. Images were analyzed by 2 neuroradiologists (D.B. and F.B.) blinded to clinical and laboratory findings and patients’ outcome. After separate evaluations, any disagreements were resolved by discussion and consensus. The evaluators independently and freely assessed the MRI acquisitions using both axial images and multiplanar reconstruction images and a predefined evaluation form. Brain lesion extension was specifically scored on FLAIR and DWI sequences by summing up the individual score for each brain hemisphere, including 5 cortical lobes (frontal, parietal, temporal, occipital, and insula) and the thalamus. A binarized score of 0 was assigned if brain parenchyma was normal, and a score of 1 was assigned if brain parenchyma depicted MRI signal abnormalities (the theoretical range of the score was 0-10) (eFigure 1 in Supplement 1). The presence of hemorrhage, detected as T2* hypointensities, and gadolinium enhancement were also assessed.

### Outcomes Assessments

The functional outcome was graded at 90 days after ICU admission using the Modified Rankin Scale (mRS).^24,25^ The mRS measures the degree of disability or dependence in the daily activities of brain-injured patients on a scale of 0 to 6, with lower scores denoting fewer disabilities or less dependence. A systematic evaluation with key criteria was made (P.J. and R.S.) on the basis of available follow-up consultation records and/or information given by physicians. A poor functional outcome at 90 days was defined by a score on the mRS of 3 to 6 (indicating moderate-to-severe disability or death). A good functional outcome at 90 days was defined by a score on the mRS of 0 to 2 (indicating a slight disability or no symptoms at all). Patients who were discharged home with functional independence before day 90 were considered to have a good functional outcome.

### Statistical Analysis

The patients’ characteristics were described as counts and frequencies for categorical variables and median (interquartile range [IQR]) for quantitative variables. Univariable comparisons between subgroups were performed using the Mann-Whitney *U* test for continuous variables and Fisher exact test for categorical variables. Univariable and multivariable analyses were used to identify radiological factors associated with poor outcome at day 90. Variables associated with the outcome in univariable analysis (*P* < .10) were entered into the multivariable model. The collinearity between variables was tested. We applied a backward elimination method, which consists of including all the chosen variables then gradually eliminating those that were nonsignificant. We performed internal validation by using the Hosmer-Lemeshow test (χ^2^ goodness of fit) with a 0.75 threshold. To extract additional clinically relevant information, we used an ensemble of supervised hierarchical classifiers methods, including Classification and Regression Trees (CART), which were applied to the variables previously selected for the multivariable analysis. The advantage of this approach is to describe the means of the distribution of the population in homogeneous groups according to 90-day survival and the clinical and radiological covariates selected from the multidimensional analysis. The CART method is based on binary recursive partitioning. The 3 basic steps of CART are as follows: first, the overall study group is split into 2 subgroups using the factor most associated with the outcome; second, this splitting into 2 is repeated within the subgroups until no further significant splits are found or the subgroups become too small; and third, the results are displayed in a binary trees structure, which, in a final step, is pruned as necessary. The final model was validated by using independent samples (80 learning samples and 58 test samples). Missing data were imputed with the median and the mode for quantitative and qualitative variables, respectively. All tests were 2-sided and were appropriately corrected for multiple comparisons; *P* < .05 was considered statistically significant. All analyses were performed using SPSS1 statistical software version 23.0 (IBM) and R statistical software version 3.5.2 (R Project for Statistical Computing). Data analysis was performed from January to April 2020.

## Results

Overall, 138 patients (median [IQR] age, 62.6 [54.0-72.0] years; 75 men [54.3%]) with a median (IQR) GCS score of 9 (6-12) at admission were studied. The median (IQR) delay between ICU admission and MRI acquisition was 1 (1-7) day. Among the 47 ICUs (259 patients) participating in the original study,^17^ investigators from 34 ICUs (194 patients) agreed to participate in this study, and we collected Digital Imaging and Communication in Medicine images from 174 patients with severe HSE who met the inclusion criteria (eFigure 2 in Supplement 1). After excluding patients who did not have MRI acquisitions before 30 days from ICU admission, patients who had poor-quality brain images, patients with associated neurological diseases, and patients lost to follow-up at 90 days, our final cohort consisted of 138 patients with confirmed severe HSE.

### Clinical and Laboratory Characteristics

Characteristics of patients are described in Table 1 and in eTable 1 in Supplement 1. HSV type 1 was responsible for HSE in 118 of 121 cases (97.5%). Overall, 21 of 137 patients (15.3%) were immunocompromised, 18 of 137 (13.1%) had diabetes, and 20 of 137 (14.6%) had chronic alcohol abuse. Functional status before ICU admission was good (ie, Knaus class A or B) in 96.4% of cases (133 of 138 patients). The main reasons for ICU admission were coma (GCS score <8) in 46 of 131 cases (35.1%) and seizures in 54 of 138 cases (39.1%). The median (IQR) body temperature at admission was 38.7 °C (38.1-39.2 °C), the median (IQR) GCS score was 9 (6-12), and focal signs were observed in 38 of 138 cases (27.5%). The median (IQR) time between first neurological symptoms and lumbar puncture was 1 (0-3) day. CSF analysis identified moderately elevated CSF leukocyte counts (median [IQR], 47 [13-160] cells/μL) with predominant lymphocytes (median [IQR], 68% [25%-220%]; to convert to proportion of 0.1, multiply by 0.01) and mildly elevated protein levels (median [IQR], 0.67 [0.49-0.96] g/dL; to convert to grams per liter, multiply by 10).

**Table 1.** Patients’ Demographic and Clinical Characteristics at ICU Admission

Characteristic	Patients, No./Total No. (%)^a^			*P* value
	Total population (N = 138)	mRS score		
		0-2 (n = 43)	3-6 (n = 95)	
Age, median (IQR), y	62.6 (54.0 to 72.0)	57.6 (42.0 to 68.0)	64.6 (5.6 to 73.6)	.005^b^
Sex				
Male	75/138 (54.3)	27/43 (62.8)	48/95 (50.5)	.20
Female	63/138 (45.7)	16/43 (37.2)	47/95 (49.5)	
Coexisting conditions				
Knaus class A or B^c^	133/138 (96.4)	43/43 (100)	90/95 (94.0)	.32
Diabetes	18/137 (13.1)	6/43 (14)	12/94 (12.8)	>.99
Alcohol abuse	20/137 (14.6)	4/43 (9.3)	16/94 (17.0)	.30
Epilepsy	1/138 (0.7)	0/43	1/95 (1.1)	>.99
Immunocompromised	21/137 (15.3)	4/43 (9.3)	17/94 (18.0)	.22
Autoimmune disease	5/137 (3.6)	0/43	5/95 (5.3)	.32
Corticosteroids	7/137 (5.1)	2/43 (4.7)	5/94 (5.3)	>.99
Hematological malignant entity	7/137 (5.1)	1/43 (2.3)	6/94 (6.4)	.43
Nonsteroidal anti-inflammatory drugs	10/137 (7.3)	3/43 (7.0)	7/94 (7.4)	>.99
Reason for ICU admission				
Altered mental status	63/138 (45.7)	18/43 (41.9)	45/95 (47.4)	.07
Seizure	54/138 (39.1)	22/43 (51.2)	32/95 (33.7)	
Other	21/138 (15.2)	3/43 (7.0)	18/95 (18.9)	
Clinical characteristics at admission				
Glasgow Coma Scale score, median (IQR)	9 (6 to 12)	10 (7 to 13)	9 (6 to 12)	.13
Glasgow Coma Scale score <8 (coma)	46/131 (35.1)	11/41 (26.8)	35/90 (38.9)	.24
Simplified Acute Physiology Score II	40/138 (29 to 55)	32 (23 to 47)	42 (32 to 58)	.001^b^
Temperature, median (IQR), °C	38.7 (38.1 to 39.2)	38.4 (38.0 to 39.0)	38.8 (38.2 to 39.2)	.10
Fever (temperature ≥38.3 °C)	87/129 (67.4)	22/39 (56.4)	65/90 (72.2)	.10
Delay ICU admission-initiation of acyclovir, median (IQR), d	0 (−1 to 0)	0 (−2 to 0)	0 (−1 to 0)	.63
ICU stay				
Seizures	97/138 (70.3)	31/43 (71.1)	66/95 (69.4)	.84
Status epilepticus	63/138 (45.6)	18/43 (41.8)	45/95 (47.3)	.59
Focal signs	38/138 (27.5)	5/43 (11.6)	33/95 (34.7)	.006^b^
Aspiration pneumonia	46/138 (33.3)	9/43 (20.9)	37/95 (38.9)	.04^b^
Invasive mechanical ventilation	95/137 (69.3)	22/43 (51.2)	73/94 (77.7)	.002^b^
ICU admission				
Home	7/136 (5.1)	3/43 (7.0)	4/93 (4.3)	.046^b^
Emergency department	70/136 (51.5)	28/43 (65.1)	42/93 (45.2)	
Internal medicine department	59/138 (43.4)	12/43 (27.9)	47/93 (50.5)	
Cerebrospinal fluid analysis				
Herpes simplex virus 1 genotype	118/121 (97.5)	35/36 (97.2)	83/85 (97.6)	>.99
Leukocyte count, cells/μL	47/138 (13 to 160)	74 (13 to 243)	39 (13 to 115)	.19
Lymphocyte count, %	68 (25 to 220)	93 (39 to 224)	65 (17 to 200)	.16
Protein level, g/dL	0.67 (0.49 to 0.96)	0.68 (0.49 to 0.89)	0.66 (0.49 to 0.95)	.82
Abnormal electroencephalogram findings	117/122 (95.9)	33/37 (89.2)	84/85 (98.8)	.03^b^
Brain imaging				
CT scan performed (at ICU admission)	112/136 (82.4)	36/43 (83.72)	76/93 (81.7)	>.99
Abnormal CT scan findings	35/111 (31.5)	12/36 (33.3)	23/75 (30.7)	.89
Delay admission MRI, median (IQR), d				
Hospital	3 (1 to 8)	2 (1 to 11)	4 (1.5 to 7.5)	.37
ICU	1 (0 to 7)	1 (0 to 8)	3 (0 to 7)	.57
Abnormal MRI findings	137/138 (99.3)	42/43 (97.7)	94/94 (100)	.31

Abbreviations: CT, computed tomography; ICU, intensive care unit; IQR, interquartile range; MRI, magnetic resonance imaging; mRS, Modified Rankin Scale.

SI conversion factors: To convert leukocyte count to cells ×10^9^/L, multiply by 0.001; leukocyte count percentage to proportion of 0.1, multiply by 0.01; protein level to grams per liter, multiply by10.

^a^ Not all patients had data available for every variable.

^b^ Indicates statistical significance at *P* < .05.

^c^ A good functional status prior admission was defined by Knaus class of A or B on a scale of A to D (A denotes normal health status, B denotes moderate activity limitations, C denotes several activity limitation due to chronic disease, and D denotes being bedridden).

### Neuroimaging Findings

The median (IQR) time between the patient’s ICU admission and the MRI acquisition was 1 (0-7) days (eFigure 3 in Supplement 1). The 2 neuroradiologists who performed blinded and separated MRI evaluations did not report any major disagreements. Contrast-enhanced T1-weighted images were available in 121 of 138 cases (89.6%). Abnormal FLAIR hyperintensities were seen in 135 cases (97.8%). FLAIR lesions extending into more than 3 lobes were identified in 53 patients (38.4%) (eTable 2 and eFigure 4 in Supplement 1). Bilateral damage was observed in 50 patients (36.2%), and FLAIR hyperintensities limited to only 1 hemisphere were identified in 85 patients (61.6%). Sixty-seven of 138 patients exhibited DWI hyperintensities. Extensive DWI signal abnormalities detected in more than 3 lobes were identified in 36 of 134 patients (26.9%). Thalamus abnormalities were identified in 62 of 134 patients (46.3%). Among these 62 cases of thalamic involvement, 27 were observed in the left thalamus and 38 were observed in the right thalamus. Parenchymal ipsilateral lesions were frequently associated with thalamic signal changes (ipsilateral, 61 of 62 cases [98.3%]; contralateral, 0 cases; bilateral, 2 of 134 cases [1.4%]). It should be noted that 45 of 62 patients (72.3%) with thalamus lesions had documentation of clinical seizures and/or epileptic abnormalities on electroencephalogram.

**Figure 1.**
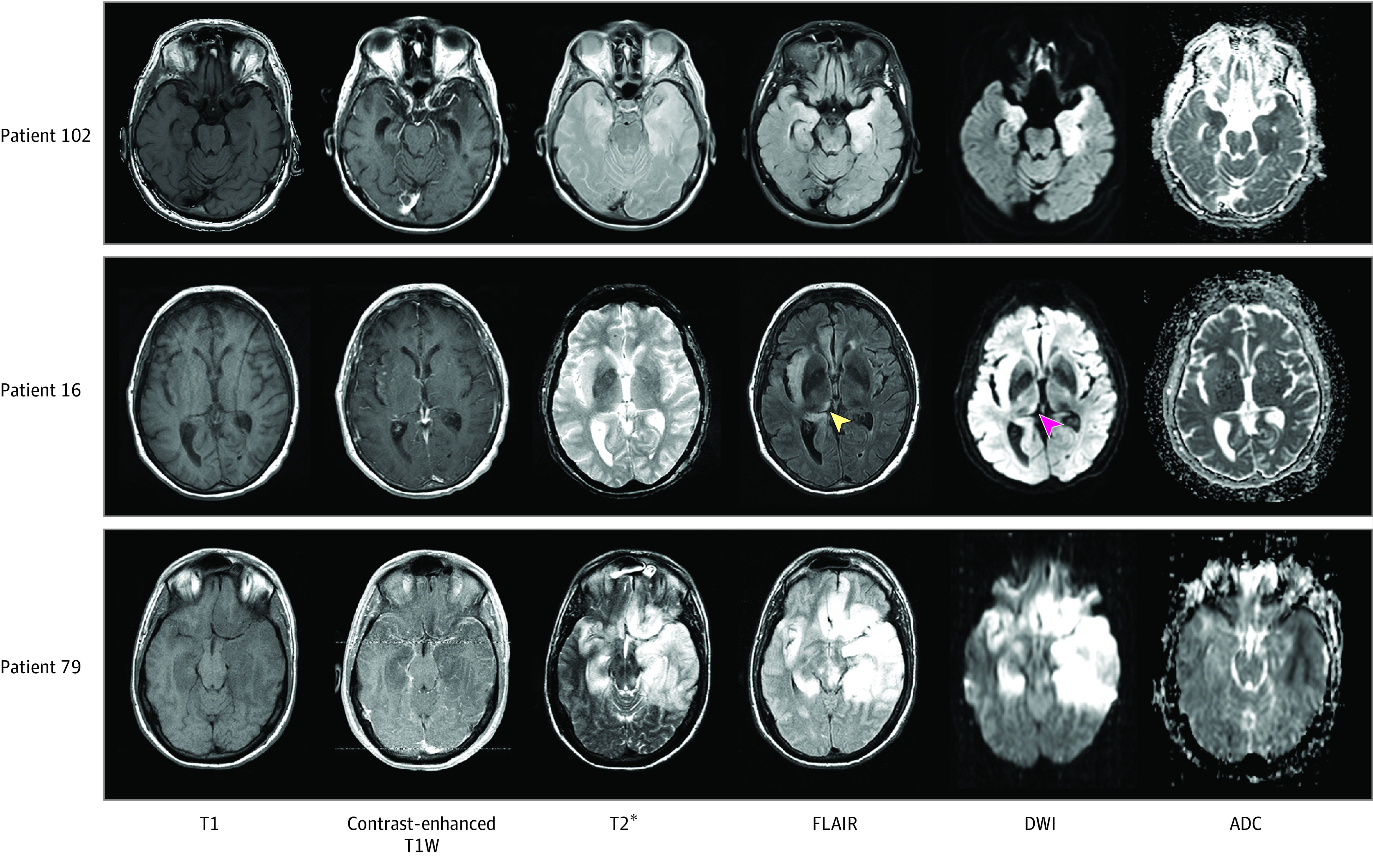
Illustrative Cases of Patients With Herpes Simplex Encephalitis Patient 102 had bilateral but asymmetrical hyperintensities in the temporal lobes seen on fluid-attenuated inversion recovery (FLAIR) images and diffusion-weighted imaging (DWI) magnetic resonance imaging sequences. Patient 16 had right thalamic hypersignal on FLAIR and DWI sequences (arrowheads), ipsilateral to the insular lesion. Patient 79 had extensive brain lesions, showing bilateral temporal, frontal, and insular signal abnormalities. ADC indicates apparent diffusion coefficient; T1W, T1-weighted.

Overall, the number of brain regions with MRI signal changes (eTable 2 in Supplement 1) was significantly higher on FLAIR sequences (median [IQR], 7 [5-9] regions) than on DWI sequences (median [IQR], 5 [3-8], regions; *P* = .02, Fisher exact test). Finally, 26 of 121 patients (21.5%) exhibited some form of leptomeningeal enhancement after contrast agent administration. Additional details and illustrations of MRI findings are provided in Figure 1 and in eTable 2 in Supplement 1.

### MRI Findings and Clinical Outcomes

**Figure 2.**
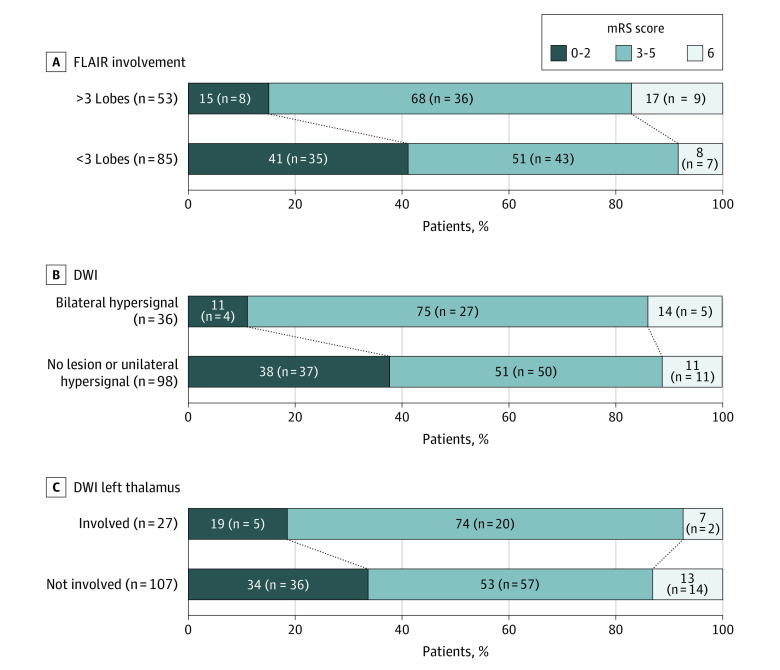
Distribution of Modified Rankin Scale (mRS) at Day 90 According to Magnetic Resonance Imaging Data On the mRS, a score of 0 to 2 indicates a good outcome, a score of 3 to 5 indicates severe disabilities, and a score of 6 indicates death. DWI indicates diffusion-weighted imaging; FLAIR, fluid-attenuated inversion recovery.

**Figure 3.**
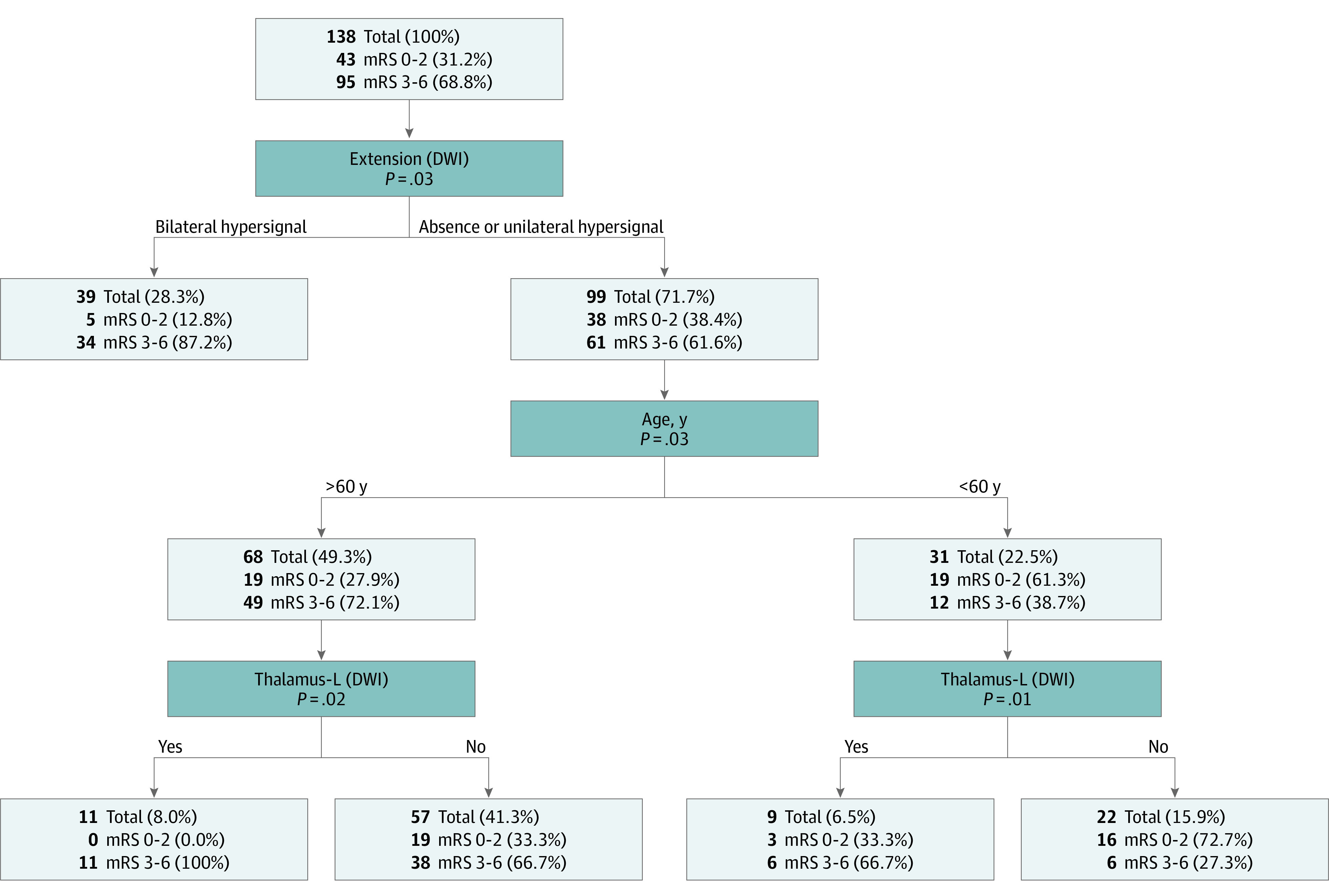
Machine Learning Model Supervised hierarchical classifier was based on both CHAID (χ^2^ automatic interaction detection) and CART (classification and regression trees) methods. The model was built and validated using independent samples (80 samples for the learning set and 58 samples for the test set). Prediction power of the model for the overall population was 75.4%. DWI indicates diffusion-weighted imaging; mRS, Modified Rankin Scale.

At 90 days, 95 patients (68.8%) had a poor outcome, including 16 deaths (11.6%). In univariable analysis, the odds of an unfavorable outcome at 90 days was higher in patients with extensive brain lesions, both on FLAIR and DWI sequences (Figure 2 and eTable 2 in Supplement 1). Moreover, we conducted a multivariable logistic regression model including 138 patients with complete data for all variables (43 favorable and 95 unfavorable outcomes) and found that FLAIR sequence signal abnormalities on more than 3 brain lobes (odds ratio [OR], 25.71; 95% CI, 1.21-554.42), age older than 60 years (OR, 7.62; 95% CI, 2.02-28.91), extensive bilateral parenchymal restricted diffusion patterns (OR, 3.17; 95% CI, 0.64-17.65), and focal diffusion signal abnormalities in the left thalamus (OR, 6.90; 95% CI, 1.12-43.00) were associated with increased odds of unfavorable outcomes (Table 2). Of note, among nonradiological variables, direct ICU admission was also associated with a better prognosis. Finally, using predictive modeling machine learning methods, we confirmed previous multivariable analyses and were able to integrate relevant data in a decision tree (Figure 3). Two main results were identified by this approach: first, the detection of bilateral DWI abnormalities was associated with worse functional prognosis (34 of 39 patients [87.2%] with bilateral abnormalities had poor outcomes; *P* = .03, Fischer exact test); second, among patients without bilateral diffusion abnormalities (ie, absence of abnormality or unilateral hypersignal), DWI hyperintensities in the left thalamus were associated with poor outcome, particularly in older patients (11 of 11 of patients aged >60 years [100%] had left thalamus abnormalities) and those with left thalamus restricted diffusion (*P* = .02, Fisher exact test).

**Table 2.** Multivariable Analysis of Factors Associated With Poor Functional Outcome^a^

Factor	OR (95% CI)	*P* value
>3 Lobes involved on fluid-attenuated inversion recovery sequence	25.71 (1.21-554.42)	.04^b^
Age >60 y	7.62 (2.02-28.91)	.002^b^
Hypersignal in left thalamus	6.90 (1.12-43.00)	.04^b^
Simplified Acute Physiology Score >34	3.91 (1.31-11.81)	.02^b^
Bilateral lesions on diffusion-weighted imaging sequence	3.17 (0.64-17.65)	.19
Direct emergency department admission^c^	0.30 (0.17-0.97)	.045^b^
Abnormalities in right thalamus on T2*-weighted sequences	0.21 (0.41-1.02)	.05

Abbreviation: OR, odds ratio.

^a^ A logistic regression model was applied. Area under the curve was 0.87 (95% CI, 0.79-0.93). Goodness of fit (Hosmer and Lemeshow test) was 0.75. Overall, 81.4% of cases were correctly classified.

^b^ Indicates statistical significance at *P* < .05.

^c^ A direct admission was defined as straightforward ICU admission from the emergency department.

## Discussion

In this large, multicenter cohort study of 138 critically ill adult patients with HSE, we observed a high disability burden, with 95 of 138 patients (68.8%) remaining functionally dependent at 90 days. Also, we observed a high mortality rate (11.6%), similar to those reported in previous studies of critically ill adult patients with HSE.^8^ To our knowledge, we have demonstrated for the first time in adults patients with HSE that brain MRI data collected during the first month after ICU admission provide relevant information regarding patients’ functional outcomes at 90 days. In agreement with our study hypothesis, MRI data, both in terms of brain lesion extension and critical focal MRI signal abnormalities, were associated with neurological outcomes.

Histopathologically, HSV infection is a fulminant necrotizing meningoencephalitis associated with edema, necrosis, hemorrhage, and encephalomalacia. Brain MRI provides accurate in vivo assessment of these pathophysiological processes. We observed that the total amount of neuroinflammatory lesions, which were mainly identified by FLAIR acquisitions, was significantly associated with patient’s functional outcome. In line with a previous report,^17^ a threshold of more than 3 brain lobes with lesions on FLAIR images (eFigure 4 in Supplement 1) was associated with the patient’s disability rating at 90 days. In addition, as previously suggested by small reports on HSE,^14,26^ DWI sequences, which reflect the molecular motion of water within the tissue, were able to identify cytotoxic and vasogenic edema lesions with interesting performances for brain lesion assessment and early diagnosis. Actually, in our cohort, DWI anomalies were a turning point regarding the patient’s functional outcome, because bilateral DWI parenchymal lesions were significantly associated with worse outcomes. It is worth noting that brain hemorrhage and blood-brain barrier disruption (detected by T2*-weighted and contrast-enhanced T1-weighted sequences, respectively) were not associated with functional outcomes.

Only scarce data exist on detailed anatomical MRI analysis of different brain substructures in patients with HSE.^10,27^ Typically, HSE causes selective damage mainly to the mesial temporal lobe’s structures, including the hippocampus.^28^ Interestingly, using a systematic whole-brain assessment of HSE’s impact, we have observed frequent thalamic involvement in severe HSE cases. This result is in contrast with previous reports^13^ suggesting that thalamic involvement is rare in patients with HSE and should make us consider alternative diagnoses in cases of encephalitis. When we specifically focused on the potential clinicopathological associations between thalamic and brain cortical lateralized MRI abnormalities, we observed that all the lateralized thalamic involvements were associated with ipsilateral cortical hemispheric lesions. We think that this finding supports the hypothesis of a diaschisis phenomenon (ie, a lesion in 1 brain region produces functional or structural impairment in a distant but interconnected brain region). Further research based on fine-grained MRI quantitative methods should be conducted to investigate this finding. It should be noted that univariable, multivariable, and machine learning analytical methods congruently identified a significant association between left thalamic HSE-related lesions and patients’ 90-day neurological outcomes. It can be hypothesized that this result might be associated with brain hemispheric dominance. However, the retrospective nature of our study did not allow us to test hemispheric dominance. Future studies should be conducted to investigate the association between lateralized thalamic lesions induced by HSE and to explore the clinical usefulness of this radiological pattern to estimate severe HSE functional outcomes.

To our knowledge, there are no solid data about HSV infection–related deep gray matter neurotropism in humans. As an alternative explanation, we suggest that thalamus signal abnormalities might reflect structural changes induced by uncontrolled epilepsy^29^ because 72.3% of patients with thalamic lesions in our cohort had epilepsy during their ICU stay.

### Strengths and Limitations

The main strengths of our study are the inclusion of a relatively large cohort, compared with previous studies, the standardized assessment of functional outcome, and a detailed blinded and independent evaluation of the radiological findings. It is worth noting that our results were consistent across different statistical approaches (ie, logistic regression and CART). This strengthens the reliability of our findings. To our knowledge, this study is the largest detailed description of MRI findings in critically ill patients with HSE with a definite outcome.

Our results must be interpreted with caution and a number of limitations should be borne in mind. The first is related to the study’s retrospective design and a lengthy study inclusion period, extended back to 2007 and prolonged over a 12-year period. Therefore, we cannot eliminate changes in neuroimaging procedures or clinical care across this period. Hence, prospective validation of these findings is certainly needed. A second limitation is that the limited sample size limits the generalizability of our finding and might be responsible for the reported modest *P* value associations and broad calculated 95% CIs for ORs. Consequently, the reported evidence requires confirmation from large-scale trials with strict recruitment criteria. A third limitation is that the patients lost to follow-up may be systematically different from the ones who remained in the study. Fourth, it can be argued that advanced MRI morphometric methods could be better suited to assess brain integrity than standard MRI in this setting. We think that our study might pave the way for future HSE studies that will specifically investigate the clinical relevance of these innovative neuroimaging techniques.

## Conclusions

In this cohort study of patients with HSE, the presence of extensive MRI lesions, on both FLAIR and DWI sequences, was independently associated with poor functional outcome at 90 days. Thalamic DWI hyperintensities were frequently observed and were associated with poor prognosis, mainly in older patients. Future studies should focus on prospectively testing the prognostic value of these neuroimaging markers. The poor outcome that we report emphasizes the importance of further research to design and validate more personalized intervention strategies for these patients, which will be at least in part built upon brain MRI data.

## Notes

Supplement 1. **eFigure 1.** Anatomical Atlas Used for Radiological Analysis **eFigure 2.** Study Flowchart **eTable 1.** Patient’s Characteristics at ICU Admission **eFigure 3.** Scattergram of Delays Between ICU Admission and MRI Acquisition **eTable 2.** MRI Collected Data **eFigure 4.** Lesion Load

Supplement 2. ENCEPHALITICA Consortium Group Members
