# Supplementary material for: Assessment of Magnetic Resonance Imaging Changes and Functional Outcomes Among Adults With Severe Herpes Simplex Encephalitis
Source: JAMA Netw Open. 2021 Jul 27;4(7):e2114328. doi: 10.1001/jamanetworkopen.2021.14328 (PMC8317014; doi:10.1001/jamanetworkopen.2021.14328)
Supplement: Supplement 1. — eFigure 1. Anatomical Atlas Used for Radiological Analysis eFigure 2. Study Flowchart eTable 1. Patient’s Characteristics at ICU Admission eFigure 3. Scattergram of Delays Between ICU Admission and MRI Acquisition eTable 2. MRI Collected Data eFigure 4. Lesion Load [file jamanetwopen-e2114328-s001.pdf]

Supplemental Online Content

Sarton B, Jaquet P, Belkacemi D, et al; ENCEPHALITICA Consortium. Assessment of magnetic resonance imaging changes and functional outcomes among adults with severe herpes simplex encephalitis. *JAMA Netw Open*. 2021;4(7):e2114328. doi:10.1001/jamanetworkopen.2021.14328

eFigure 1. Anatomical Atlas Used for Radiological Analysis
eFigure 2. Study Flowchart
eTable 1. Patient's Characteristics at ICU Admission
eFigure 3. Scattergram of Delays Between ICU Admission and MRI Acquisition
eTable 2. MRI Collected Data
eFigure 4. Lesion Load

This supplemental material has been provided by the authors to give readers additional information about their work.

© 2021 Sarton B et al. *JAMA Network Open.*

eFigure 1. Anatomical Atlas Used for Radiological Analysis

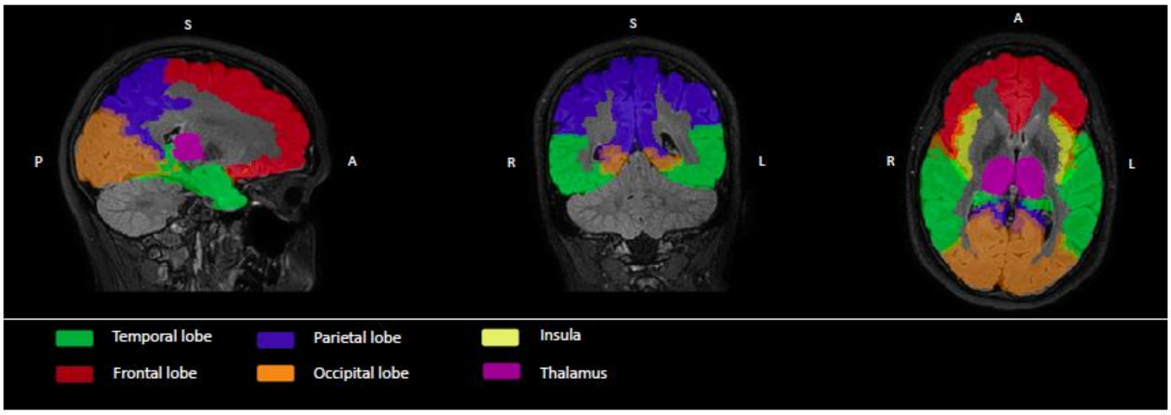

Template in FLAIR sequences from the cohort study. From left to right: sagittal, frontal and coronal planes. Coloured layers of anatomical lobes have been applied. A = Anterior. L = Left. R = Right. S = Superior.

© 2021 Sarton B et al. *JAMA Network Open.*

eFigure 2. Study Flowchart

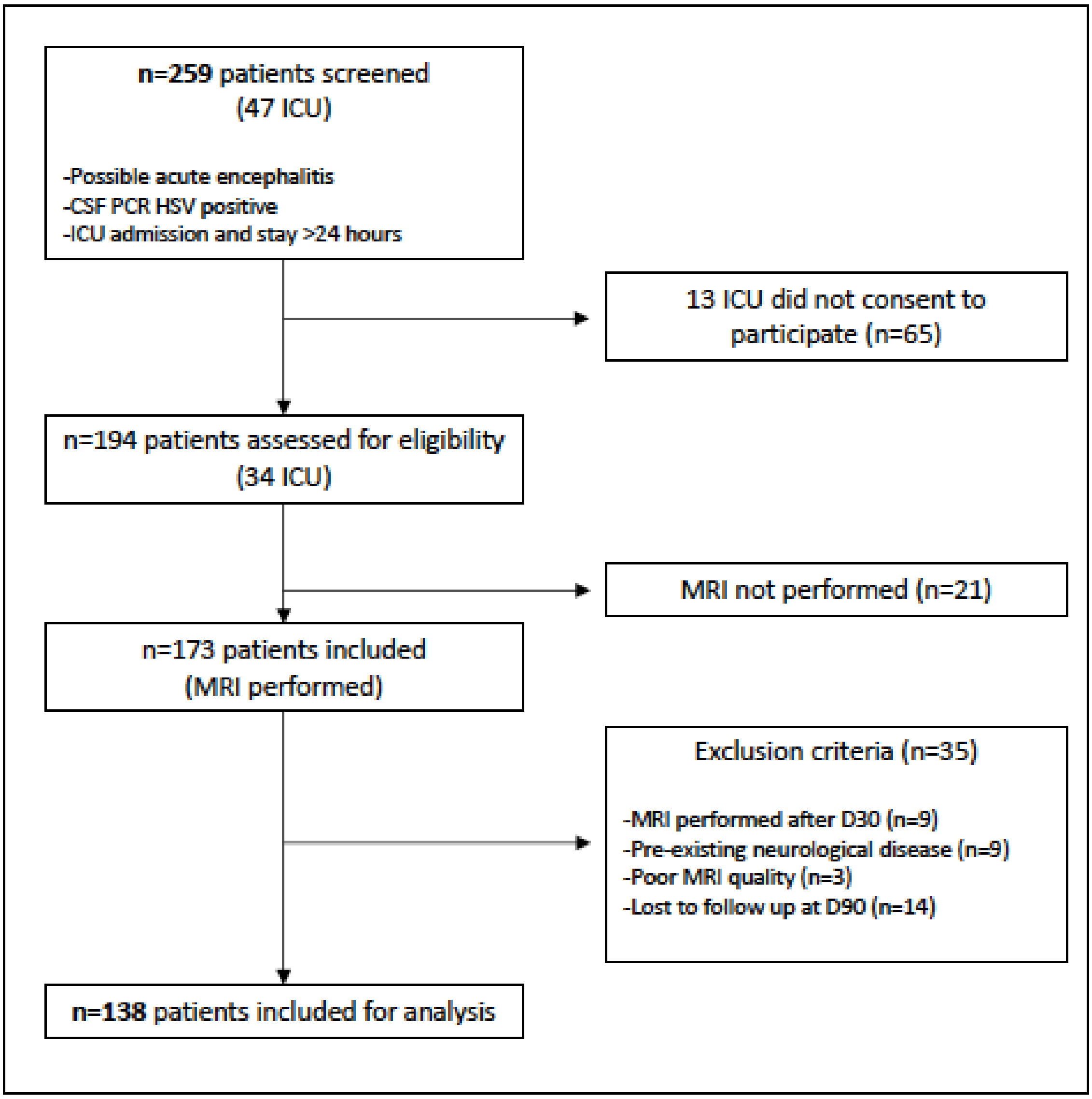

259 patients admitted in ICU for confirmed severe herpes simplex encephalitis, were screened between January 2006 and December 2016. Among them, 138 patients were included for final analysis after applying inclusion and exclusion criteria. Abbreviations: CSF = cerebrospinal fluid. HSV = Herpes Simplex Virus. ICU = Intensive Care Unit. MRI = Magnetic Resonance Imaging.

© 2021 Sarton B et al. *JAMA Network Open.*

eTable 1. Patient's Characteristics at ICU Admission: Whole Dataset (n=259)

	**Whole dataset (n=259)**	**Used for analysis (n=138)**
**Demographics**		
Age (years)	64 (54 to 73)	62·6 (54 to 72)
Male sex	132 (51%)	75 (54·3%)
**Coexisting conditions**		
Knaus score A or B	254/257 (95%)	133 (96·4%)
Diabetes	36/256 (14%)	18/137 (13·1%)
Alcohol abuse	33/256 (13%)	20/137 (14·6%)
Epilepsy	9/257 (4%)	1 (0·7%)
Immunocompromised	49/256 (19%)	21/137 (15·3%)
**Reason for ICU admission**		
Altered mental status	128 (49·4%)	63 (45·7%)
Seizure	65 (25·1%)	54 (39·1%)
Other (mainly respiratory failure)	66 (25·5%)	21 (15·2%)
**Clinical characteristics at admission**		
Glasgow coma scale (GCS)	9 (6 to 12)[f]	9 (6 to 12)[a]
GCS<8 (indicating coma)	89/243 (34%)	46/131 (35·1%)
Temperature (°C)	38·7 (38·1 to 39·2)[g]	38·7 (38·1 to 39·2)[b]
Fever (temperature≥ 38·3 °C)	165/241 (69%)	87/129 (67·4%)
Delay ICU admission-initiation of acyclovir (days)	0 [0 - 1]	0 [-1 - 0]
**ICU stay**		
Seizures in ICU	168/258 (64,9%)	97 (70·3%)
Status epilepticus in ICU	95/258 (36,8%)	63 (45·6·%)
Focal signs	68/258 (26,3%)	38 (27·5%)
Aspiration pneumonia	72/258 (29%)	46 (33·3%)
Invasive mechanical ventilation	166 (62%)	95/137 (69·3%)
Direct ICU admission	136/257 (53%)	77/136 (56·5%)
**CSF analysis**		
HSV 1 genotype	205/215 (95%)	118/121 (97·5%)
Leukocyte count (/mm^3)	50 (12 to 140)[h]	47 (13 to 160)[c]
Lymphocyte count (%)	86 (61 to 96)[i]	68 (25 to 220)[d]
Protein level (g/l)	0·7 (0·5 to 1·1)[j]	0·67 (0·49 to 0·96)[e]
**EEG**		
Abnormal EEG	218/226 (96%)	117/122 (95·9%)
**Brain imaging**		
Delay hospital admission-MRI (days)	3[1-9]	3 [1-8]
Delay ICU admission-MRI (days)	1[0-7]	1[0-7]
Abnormal MRI	221/223 (98%)	137/138 (99·3%)

Results are expressed as median (IQR) for continuous data and or n (%) evaluable for categorial data. A good functional status prior admission was defined by KNAUS score of A or B. Immunodepression was defined as the use of long term (> 3 months) steroids, the use of other immunosuppressant drugs, solid organ transplantation, solid tumours requiring chemotherapy in the last 5 years, haematological malignancy, or AIDS. Direct ICU admission means patient coming from home or emergency unit (not from internal medicine department). Abnormal EEG including epileptiform pattern, asymmetry; triphasic waves, spike waves; slow activity, periodic lateralized epileptiform discharge (PLEDS). mRS = modified Rankin Scale. ICU = Intensive care unit. GCS = Glasgow Coma Scale. CSF = Cerebrospinal fluid. HSV = Herpes simplex virus. EEG = electroencephalogram. MRI = Magnetic resonance imaging. SAPS=Simplified acute physiology score.

© 2021 Sarton B et al. *JAMA Network Open.*

eFigure 3. Scattergram of Delays Between ICU Admission and MRI Acquisition

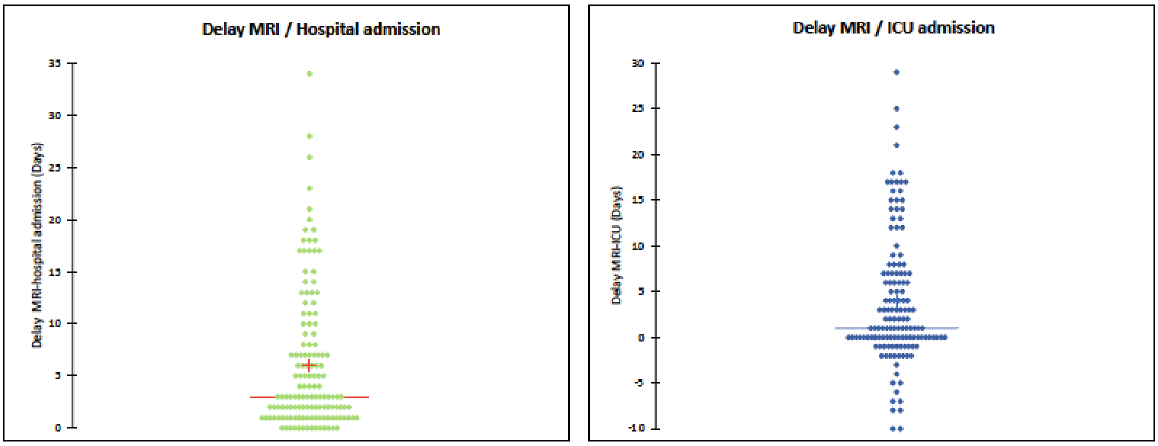

Medians are represented with plain a bar and average values with a cross. Median time between ICU admission and MRI was 1[0-7] days. ICU=Intensive care unit.

© 2021 Sarton B et al. *JAMA Network Open.*

eTable 2. MRI Collected Data

	Total population	mRS 0-2	mRS 3-6	P value
**T2-FLAIR**				
**Hypersignal (FLAIR)**				
Right Insula	72 (52·2%)	19 (44·2%)	53 (55·8%)	0·269
Left Insula	77 (55·8%)	19 (44·2%)	58 (61·1%)	0·095
Right temporal lobe	86 (62·3%)	25 (58·1%)	61 (64·2%)	0·570
Left temporal lobe	81 (58·7%)	21 (48·8%)	60 (63·2%)	0·136
Right frontal lobe	70 (50·7%)	19 (44·2%)	51 (53·7%)	0·359
Left frontal lobe	59 (42·8%)	11 (25·6%)	48 (50·5%)	0·008*
Right thalamus	59 (42·8%)	18 (41·9%)	41 (43·2%)	0·999
Left thalamus	42 (30·4%)	10 (23·3%)	32 (33·7%)	0·237
**Lateralization (FLAIR)**				
No lesion	3 (2·2%)	2 (4·7%)	1 (1·1%)	0·036*
Unilateral	85 (61·6%)	32 (74·4%)	53 (55·8%)	
-Right	42 (30·4%)	18 (41·9%)	24 (25·3%)	
-Left	43 (31·2%)	14 (32·6%)	29 (30·5%)	
Bilateral	50 (36·2%)	9 (20·9%)	41 (43·2%)	
**Lesion load (FLAIR)**				
Number of lobes involved (0 to 10)	3 (2 to 5)	3 (2 to 3)	3 (3 to 5)	0·0004*
0 to 3 lobes	85 (61·6%)	35 (81·4%)	50 (52·6%)	0·005*
4 to 6 lobes	44 (31·9%)	7 (16·3%)	37 (38·9%)	
7 to 10 lobes	9 (6·5%)	1 (2·3%)	8 (8·4%)	
MRI brain lesions > 3 lobes	53 (38·4%)	8 (18·6%)	45 (47·4%)	0·001*
**DWI MRI**				
**Hypersignal (DWI)**				
Right Insula	64 (47·8%)	16 (39%)	48 (51·6%)	0·194
Left Insula	61 (45·5%)	13 (31·7%)	48 (51·6%)	0·039
Right temporal lobe	67 (50%)	17 (41·5%)	50 (53·8%)	0·260
Left temporal lobe	64/134 (47·8%)	15/41 (36·6%)	49/93 (52·7%)	0·094
Right frontal lobe	52/134 (38·8%)	12/41(29·3%)	40/93 (43%)	0·178
Left frontal lobe	37/134 (27·6%)	6/41 (14·6%)	31/93 (33·3%)	0·035
Right thalamus	40/134 (29·8%)	16/42 (38·1%)	24/93 (25·8%)	0·159
Left thalamus	28/134 (20·7%)	4/42 (9·5%)	24/93 (25·8%)	0·038
**Lateralization (DWI)**				
No lesion	16 (11·9%)	9 (22%)	7 (7·5%)	0·007*
Unilateral	82 (61·2%)	28 (68·3%)	54 58·1%)	
-Right	41 (30·6%)	15 (36·6%)	26 (28%)	
-Left	41 (30·6%)	13 (31·7%)	28 (30·1%)	
Bilateral	36 (26·9%)	4 (9·8%)	32 (34·4%)	
**Lesion load (DWI)**				
Number of lobes involved (0 to 10)	3 (2 to 4)	2 (1 to 3)	3 (2 to 5)	0·0005*
0 to 3 lobes	98 (73·1%)	37 (90·2%)	61 (65·6%)	0·01*
4 to 6 lobes	30 (22·4%)	4 (9·8%)	26 (28%)	
7 to10 lobes	6 (4·5%)	0 (0%)	6 (6·5%)	
**T2***				
T2* (n=111)	24 (21·6%)	11 (31·4%)	13 (17·1%)	0·66
**T1- GADOLINIUM**				
Leptomeningeal enhancement (n=121)	26 (21·5%)	9 (25%)	17 (20%)	0·54

Overall, 138 MRI were analysed. Signal abnormalities were counted as present / absent per lobe (right or left), unless otherwise mentioned. Abbreviations: mRS = modified Rankin Scale, FLAIR = Fluid-Attenuated Inversion Recovery.

© 2021 Sarton B et al. *JAMA Network Open.*

eFigure 4. Lesion Load

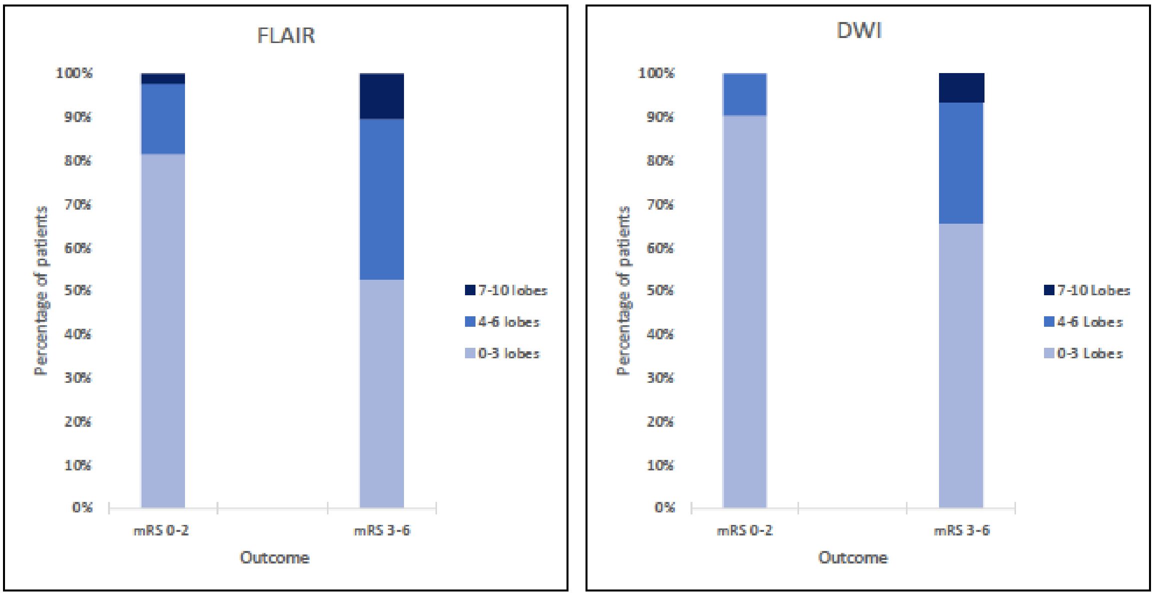

Stacked bars representing the number of lobes involved on early MRI in the groups "favourable outcome" (mRS: 0-2) and "unfavourable outcome" (mRS 3-6). FLAIR: $p<0{\cdot}005$. DWI: $p<0{\cdot}01$. DWI = Diffusion weighted imaging. FLAIR = Fluid-attenuated inversion recovery.

© 2021 Sarton B et al. *JAMA Network Open.*
