# Supplementary material for: Assessment of Magnetic Resonance Imaging Changes and Functional Outcomes Among Adults With Severe Herpes Simplex Encephalitis
Source: JAMA Netw Open. 2021 Jul 27;4(7):e2114328. doi: 10.1001/jamanetworkopen.2021.14328 (PMC8317014; doi:10.1001/jamanetworkopen.2021.14328)
Supplement: Supplement 2. — ENCEPHALITICA Consortium Group Members [file jamanetwopen-e2114328-s002.pdf]

***Group Name(s): ENCEPHALITICA Consortium**

***First Name and Middle Initial(s)**	**Last Name**	***Suffix (eg, Jr, III)**	Academic Degrees	Institution	Location (city, state/province, country)	Role or Contribution, eg, chair, principal investigator	Group (if more than 1 Group listed in the byline) and/or Subgroup (eg, Steering Committee)
**Pierre**	**Jaquet**		MD	Department of intensive care medicine and infectious diseases, Bichat-Claude Bernard University Hospital, A	Paris, France	Data collection	ENCEPHALITICA study group
**Etienne**	**de Montmollin**		MD	Department of intensive care medicine and infectious diseases, Bichat-Claude Bernard University Hospital, A	Paris, France	Data collection	ENCEPHALITICA study group
**Claire**	Dupuis		MD	Department of intensive care medicine and infectious diseases, Bichat-Claude Bernard University Hospital, A	Paris, France	Data collection	ENCEPHALITICA study group
**Mikael**	Alves		MD	Polyvalent intensive care unit, Poissy–Saint-Germain-en-Laye Hospital	Poissy, France	Data collection	ENCEPHALITICA study group
**Laurent**	Argaud		MD, PhD	Medical intensive care unit, CHU Edouard Herriot	Lyon, France	Data collection	ENCEPHALITICA study group
Pierre	Bailly		MD	medical Intensive Care Unit, La Cavale Blanche University Hospital	Brest, France	Data collection	ENCEPHALITICA study group
François	Barbier		MD	medical intensive care unit, La Source hospital,	Orleans, France	Data collection	ENCEPHALITICA study group
Lila	Bouadma		MD, PhD	department of intensive care medicine and infectious diseases, Bichat-Claude Bernard University Hospital, AP-HP	Paris, France	Data collection	ENCEPHALITICA study group
**Noelle**	Brulé		MD	medical intensive care unit, Nantes University Hospital,	Nantes, France	Data collection	ENCEPHALITICA study group
Fabrice	Brunel		MD	medical intensive care unit, André Mignot Hospital,	Versailles, France	Data collection	ENCEPHALITICA study group
Russell	**Chabanne**		MD	department of perioperative medicine, Clermont-Ferrand university hospital,	Clermont-Ferrand, France	Data collection	ENCEPHALITICA study group
**Marie**	**Conrad**		MD	medical intensive care unit, CHU de Nancy	Nancy, France	Data collection	ENCEPHALITICA study group
**Daniel**	Da Silva		MD	medical intensive care unit, Delafontaine Hospital,	Saint-Denis, France	Data collection	ENCEPHALITICA study group
Frederic	Dailler		MD	department of anesthesiology and intensive care medicine, Hôpital Pierre Wertheimer,	Lyon, France	Data collection	ENCEPHALITICA study group
**Delphine**	**Daubin**		MD	medical intensive care Unit, Montpellier university hospital,	Montpellier, France	Data collection	ENCEPHALITICA study group
**Sophie**	**Demeret**		MD	neurological intensive care unit, CHU Pitié Salpétrière,	Paris, France	Data collection	ENCEPHALITICA study group
**Nicolas**	**Lerolle**		MD, PhD	medical intensive care unit, CHU Angers, Angers university hospital	Angers, France	Data collection	ENCEPHALITICA study group
Julien	Marechal		MD	medical intensive care unit, Centre Hospitalier Universitaire de Poitiers	Poitiers, France	Data collection	ENCEPHALITICA study group
**Bruno**	**Mourvillier**		MD	medical intensive care unit, CHU de Reims	Reims, Fra,ce	Data collection	ENCEPHALITICA study group
**Ahmed**	**El Kalioubi**		MD	medical intensive care unit, Roger Salengro University Hospital,	Lille, France	Data collection	ENCEPHALITICA study group
**Benjamine**	Sarton		MD	intensive care unit, Purpan University Hospital	Toulouse, France	Data collection	ENCEPHALITICA study group
Stein	Silva		MD, PhD	intensive care unit, Purpan University Hospital	Toulouse, France	Data collection	ENCEPHALITICA study group

Supplemental Online Content: Nonauthor Collaborators

*Indicates required information. Only first name, last name, and suffix will appear in PubMed.

*First Name and Middle Initial(s)	Last Name	*Suffix (eg, Jr, III)	Academic Degrees	Institution	Location (city, state/province, country)	Role or Contribution, eg, chair, principal investigator	Group (if more than 1 Group listed in the byline) and/or Subgroup (eg, Steering Committee)
**Romain**	Sonneville		MD, PhD	department of intensive care medicine and infectious diseases, Bichat-Claude Bernard University Hospital, A	Paris, France	Data collection	ENCEPHALITICA study group
**Vincent**	**Susset**		MD	polyvalent intensive care unit,	Chambery, France	Data collection	ENCEPHALITICA study group
**Jean Marc**	Tadié		MD	intensive care medicine, CHU Pontchaillou	Rennes, France	Data collection	ENCEPHALITICA study group
Jean-Françoit	Timsit		MD, PhD	department of intensive care medicine and infectious diseases, Bichat-Claude Bernard University Hospital, A	Paris, France	Data collection	ENCEPHALITICA study group
**Michel**	Wolff		MD, PhD	neurological ICU, Hôpital Saint Anne	Paris, France	Data collection	ENCEPHALITICA study group
**Alexandre**	Lautrette		MD, PhD	medical intensive care unit, Gabriel-Montpied University Hospital	Clermont-Ferrand, France	Data collection	ENCEPHALITICA study group
Emmanuel	Novy		MD	Polyvalent Intensive Care Unit, Mercy Hospital CHR Metz-Thionville,	Ars-Laquenexy, France	Data collection	ENCEPHALITICA study group
**Bertrand**	Guidet		MD, PhD	Medical Intensive Care Unit, Hôpital Saint-Antoine	Paris, France	Data collection	ENCEPHALITICA study group
**François**	Mateos		MD	Medical Intensive Care Unit and UMR 1121, Hautepierre Hospital Strasbourg	Strasbourg, France	Data collection	ENCEPHALITICA study group
**Clément**	**Brault**		MD	Department of Intensive Care Medicine, Amiens-Picardie University Hospital,	Amiens, France	Data collection	ENCEPHALITICA study group
**Quentin**	**Maestraggi**		MD	Medical Intensive Care Unit and UMR 1121, Hautepierre Hospital Strasbourg	Strasbourg, France	Data collection	ENCEPHALITICA study group
**Keyvan**	**Razazi**		MD	Medical Intensive Care Unit, hopitaux Universitaires Henri Mondor	Créteil, France	Data collection	ENCEPHALITICA study group
**Jean-Pierre**	Quenot		MD, PhD	Medical Intensive Care Unit, Centre Hospitalo-Universitaire de Dijon	Dijon, France	Data collection	ENCEPHALITICA study group
Aurélie	Joret		MD	Medical Intensive Care Unit, CHU de Caen	Caen, France	Data collection	ENCEPHALITICA study group
Albrice	**Levrat**		MD	Intensive care unit, Centre Hospitalier Annecy-Genevois,	Annecy, France	Data collection	ENCEPHALITICA study group
Alexandre	Massri		MD	Intensive Care Unit, Centre Hospitalier Francois Mitterand	Pau, France	Data collection	ENCEPHALITICA study group
Alexandre	Robert		MD	Medical Intensive Care Unit, Hôpital de l'Archet 1, CHU de Nice	Nice,France	Data collection	ENCEPHALITICA study group
**Damien**	**Contou**		MD	Polyvalent Intensive Care Unit, Centre Hospitalier Victor Dupouy,	Argenteuil, France	Data collection	ENCEPHALITICA study group
**Jean-Paul**	Mira		MD, PhD	Medical Intensive Care Unit, Cochin University Hospital	Paris, France	Data collection	ENCEPHALITICA study group
**Stephane**	Gaudry		MD	Polyvalent Intensive Care Unit, Louis Mourier University Hospital	Colombees, France	Data collection	ENCEPHALITICA study group
**Guillaume**	Voiriot		MD, PhD	Polyvalent Intensive Care Unit, Centre Hospitalier Universitaire Tenon	Paris, France	Data collection	ENCEPHALITICA study group
**Asael**	Berger		MD	Intensive Care Unit, Centre Hospitalier de Haguenau,	Haguenau, France	Data collection	ENCEPHALITICA study group
**Vincent**	Das		MD	Medical-Surgical Intensive Care Unit, Centre Hospitalier Intercommunal André Grégoire	Montreuil, France	Data collection	ENCEPHALITICA study group

Supplemental Online Content: Nonauthor Collaborators

*Indicates required information. Only first name, last name, and suffix will appear in PubMed.

***First Name and Middle Initial(s)**	**Last Name**	***Suffix (eg, Jr, III)**	Academic Degrees	Institution	Location (city, state/province, country)	Role or Contribution, eg, chair, principal investigator	Group (if more than 1 Group listed in the byline) and/or Subgroup (eg, Steering Committee)
**Nicolas**	**Engrand**		MD	Neurosurgical Intensive Care Unit, Fondation ophtalmologique Adolphe Rotschild	Paris, France	Data collection	ENCEPHALITICA study group
**Martin**	Murgier		MD	Intensive care unit, Saint-Etienne University Hospital	Saint Etienne, France	Data collection	ENCEPHALITICA study group
**Shidasp**	**Siami**		MD	Polyvalent ICU, Centre Hospitalier Sud Essone Dourdan-Etampes-Siège,	Etampes, France	Data collection	ENCEPHALITICA study group
Sami	Hraiech		MD, PhD	Medical Intensive Care Unit, Aix-Marseille Universite, Hopital Nord	Marseille, France	Data collection	ENCEPHALITICA study group
**Eric**	**Mariotte**		MD	Medical Intensive Care Unit, Saint-Louis Hospital	Paris, France	Data collection	ENCEPHALITICA study group
**Claire**	Ragot		MD	Medical-Surgical Intensive Care Unit, Robert Boulin Hospital	Libourne, France	Data collection	ENCEPHALITICA study group
Annabelle	Stoclin		MD	Intensive Care Unit, Institut Gustave Roussy	Paris, France	Data collection	ENCEPHALITICA study group
Pierre	Trouiller		MD	Intensive Care Unit, Antoine Béclère Hospital	Clarmart, France	Data collection	ENCEPHALITICA study group
Mathieu	Schmidt		MD	Medical Intensive Care Unit, Hôpital Pitié – Salpêtrière, AP-HP	Paris, France	Data collection	ENCEPHALITICA study group
**Charline**	Sazio		MD	Medical intensive care unit, Bordeaux University Hospital	Bordeaux, France	Data collection	ENCEPHALITICA study group
